# Sorption of per- and poly-fluoroalkyl substances and their precursors on activated carbon under realistic drinking water conditions: Insights into sorbent variability and PFAS structural effects

**DOI:** 10.1016/j.heliyon.2024.e25130

**Published:** 2024-01-26

**Authors:** Mohammad Sadia, Lola Beltrán Beut, Marko Pranić, Annemarie P.van Wezel, Thomas L.ter Laak

**Affiliations:** a Institute for Biodiversity and Ecosystem Dynamics, University of Amsterdam, Science Park 904, 1098XH, Amsterdam, the Netherlands; b Environmental Technology, Wageningen University, Bornse Weilanden 9, 6708WG, Wageningen, the Netherlands; c KWR Water Research Institute, Groningenhaven 7, 3430BB Nieuwegein, the Netherlands

**Keywords:** PFAS and PFAS precursor sorption, GAC, Reactivated GAC, Sorption mechanisms, Drinking water production

## Abstract

Recent stringent drinking water quality standards create challenges for water utilities to meet these standards. Advanced treatment techniques will have to be applied on many drinking water production locations to meet these quality standards. This study investigated the sorption of per- and polyfluorinated-alkyl substances (PFAS) onto granular activated carbon (GAC). The study was performed at environmentally relevant PFAS concentrations and a realistic water-to-GAC ratio, providing a realism often overlooked in existing studies. Three different forms of GAC were evaluated, differing in micropore and mesopore structures. Tap water spiked with 5 ng/L of each of 31 PFAS was used in the sorption experiments, i.e. perfluorocarboxylic acids (C_4_–C_12_), perfluorosulfonic acids (PFSA, C_5_–C_10_) including linear and branched isomers, and three groups of PFAS precursors (per-/polyfluoroalkyl ether acids, sulfonamides, and sulfonamide acetic acids).

The three studied GAC did not exhibit distinct differences in PFAS sorption. The removal of PFAS was below 50 % for most studied PFAS, except for the short-chain PFAS precursors. Sorption was affected by both the carbon chain length and functional groups for PFAS, while this was not observed for PFAS precursors. The presence of ether linkages and sulfonamide groups notably enhanced sorption. Linear and branched PFSA demonstrated similar sorption behavior, whereas branched isomers of the sulfonamide acetic acid precursors exhibited significantly higher sorption. This indicates that sorption was determined by both hydrophobic and electrostatic interactions.

Given the relatively low PFAS removal by GAC under environmentally relevant test conditions, further improvements in sorbents are required to ensure that PFAS concentrations in produced drinking water comply with drinking water standards.

## Highlights

• Activated carbon showed limited removal of Per/Poly-fluoroalkyl Substances (PFAS) at environmentally relevant concentrations.
• Interactions between PFAS and activated carbon are explained by hydrophobic and electrostatic interactions.
• Structural features of PFAS precursors are identified as key drivers for their sorption onto activated carbon.

## Graphical abstract

**Figure:**
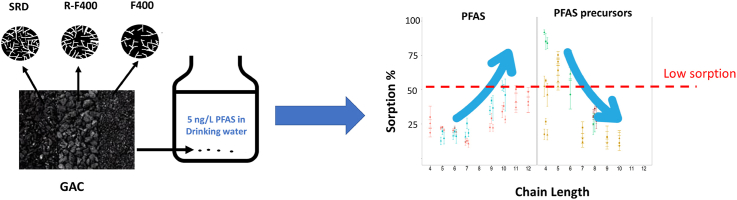

## 1 Introduction

Ensuring access to safe drinking water is a fundamental human right, yet it faces challenges due to environmental contamination and increasing water scarcity [1]. The presence of various anthropogenic contaminants poses a threat to both human and environmental health [2,3]. Among these contaminants, poly- and perfluoroalkyl substances (PFAS) are a group of synthetic chemicals widely used for their oil- and water-repellent properties [4]. PFAS enter the environment through various routes, including industrial and municipal wastewater treatment plants, landfills, recycling and incineration facilities, and even the use of consumer products [[5], [6], [7], [8]]. These substances are now ubiquitous in the environment, wildlife, and humans [[9], [10], [11], [12], [13], [14], [15]].

Drinking water is a significant route of human exposure to PFAS [16], with the imperative need to ensure its safety for human health. Various authorities are imposing increasingly stringent guidelines and recommendations regarding safe PFAS levels in drinking water and other exposure media, reflecting evolving knowledge regarding their toxicity and exposure dynamics [[16], [17], [18], [19], [20]]. Meeting these regulations presents a challenge for water treatment facilities [21,22], especially given the limitations of conventional and advanced treatment methods in effectively removing PFAS [14,23].

Granular activated carbon (GAC), a type of carbonaceous sorbent, is commonly used in drinking water treatment due to its cost-effectiveness and efficiency in removing various contaminants [24,25]. GAC primarily relies on hydrophobic and electrostatic sorption mechanisms [26,27]. With most PFAS existing in their anionic form in aquatic environments (low pKa values, typically <3 [28]), they interact electrostatically with charged surface groups on GAC. Additionally, the hydrophobic tail of PFAS results in enhanced sorption for longer chain PFAS through hydrophobic interactions [[29], [30], [31]].

Innovative forms of GAC are currently under development to enhance the removal of micropollutants from water. This is achieved by modifying the sorbent's surface chemistry and physical properties. Sorbent surface chemistry can be characterized by elemental composition [32], surface acidity and basicity [33], and point of zero charge [34]. Physical properties are summarized in terms of porosity, pore volume, and pore shape [35]. Furthermore, the sorption of PFAS and other micropollutants can be influenced by aqueous chemistry, including factors such as pH [34], co-occurring organic compounds like organic matter (OM) [36], and inorganic ions [32].

Given that guidelines for safe drinking water levels have become stricter over the past decades [37], there is an urgent need for more effective water treatment processes to ensure that PFAS concentrations in drinking water remain below these levels. Recent studies have shown that some Dutch drinking water treatment plants, which apply GAC treatment, complied with regulatory drinking water standards but were unable to meet guideline values such as the European Food Safety Authority (EFSA) recommendation for total PFAS intake TWI of 4.4 ng/kg bodyweight [14]. Previous research on PFAS sorption onto GAC had primarily focused on well-known PFAS like PFOA and PFOS at levels far above environmental concentrations (μg/L, mg/L) [38]. This highlights the pressing need to gain a deeper understanding of PFAS and PFAS precursor sorption onto GAC at concentrations within the ng/L range, given that GAC treatment is the most used advanced technology for drinking water production [24,25].

Therefore, this study aims to investigate the sorption behavior of a broad spectrum of PFAS at environment relevant concentrations (∼5 ng/L) in a real drinking water matrix using realistic water to GAC ratio. The PFAS mixture includes PFCA: perfluoro-carboxylic acids (C_4_–C_12_), PFSA: perfluoro-sulfonic acids (C_5_–C_10_), and PFAS precursor groups: per-/poly-fluoroalkyl of the ether acids (PFEA), sulfonamides, and sulfonamide acetic acids, including both branched and linear isomers for three different PFAS. Three distinct GAC sorbents were used, varying in porosity and micro- and mesopore volumes, including thermally reactivated GAC, as it is relevant to the drinking water production practices. The study aims to provide insight into the role of the chain length, functional groups, and branching tail structure of the PFAS and PFAS precursors in sorption onto GAC under realistic drinking water production conditions.

## 2 Material and methods

### 2.1 Sorbents

Three bituminous coal-based granulated activated carbon (GAC) were acquired from Calgon Carbon Corporation, Feluy, Belgium, namely Filtrasorb® 400 (F400), reactivated Filtrasorb® 400 (R–F400) and the carbon sorbent SRD. A detailed material characterization of the adsorbents, including density, micropores and mesopore volume, particle porosity, and pH point of zero charge (pH_pzc_), is provided in Table S2. Details on the method used for measuring the GAC porosity and pH_pzc_ listed in the supplementary information. All studied GAC had a similar total porosity of 50–58 %. F400 has a slightly lower mesopore volume, and is commonly used in drinking water treatment. R–F400 was provided by supplier and is F400 that was reactivated at least five times by thermal reactivation to 700 °C. The SRD sorbent is a new GAC that offers a higher percentage of mesopores without reducing the micropore volume compared to F400.

Prior to the experiment the sorbents were sieved using US standard sieves to obtain a uniform particle size of 1–2 mm, they were washed with Milli-Q water to remove fines, and dried at 150 °C for 40 min, then the dried GAC stored at room temperature until used in experiments.

### 2.2 Sorption experiment

**Fig. 1:**
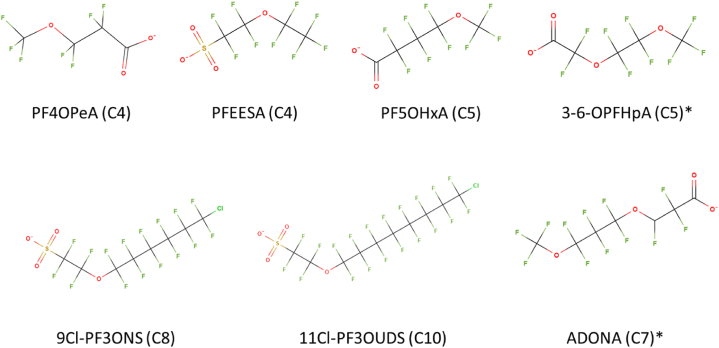
Chemical structure of the studied per-/poly-fluoroalkyl ether acids (PFEA) with different chain lengths. The * indicates PFEA with a di-ether structure.

Batch experiments were conducted using tap water from Amsterdam, the chemical analysis for the water are reported regularly by the drinking water supplier [39]. A mixture of 31 PFAS was selected as sorbates to represent a set of anionic PFAS with varying hydrophobicity (different chain length, branched and linear isomers) and types of hydrophilic head groups (carboxyl, sulfon, sulfonamide, sulfonamide acetic acid, and ether). Specifically, perfluoro-carboxylic acids (PFCA, C_4_–C_12_), perfluoro-sulfonic acids (PFSA, C_4_–C_10_), and three groups of precursors (i.e. per-/poly-fluoroalkyl of the ether acids (PFEA, Fig. 1), sulfonamides, and sulfonamide acetic acids) were selected, including branched isomers for PFHxS, EtFOSAA, and MeFOSAA (Table S1).

For the sorption experiments, 500 mL of tap water in 2 L HDPE plastic bottles were spiked with the PFAS mixture (12.5 μL; 0.2 ng/μL for each PFAS in methanol; Table S2), followed by the addition of 5.0 ± 0.1 mg of sieved GAC. The bottles were then placed in a laboratory shaker at a constant temperature of 22 ± 1 °C for specific durations (1 h, 5 h, 24 h, 96 h, 240 h, all in triplicate). After the designated time intervals, the sorbent was removed, and solid-phase extraction (SPE) was performed on the aqueous samples.

The batch experiments using GAC may not capture the full dynamics of continuous treatment systems found in real-scenario drinking water treatment plants [40,41]. However, these experiments provide valuable insights into PFAS sorption onto GAC. Water to GAC ratio was chosen based on real scenario application of powdered activated carbon (PAC) in the drinking water treatment plant (average 3–7 mg/L) [42].

### 2.3 Chemical analysis

Aqueous phase samples were extracted using solid phase extraction (SPE) and analyzed for 28 PFAS using liquid chromatography coupled with high resolution mass spectrometry. The complete list of analytes and details of the analytical methods and chemicals used are provided in the supplementary information (Tables S1 and S3).

### 2.4 Quality control

The sorption experiment was conducted in triplicate, and the relative standard deviation (RSD%) of the triplicate analyses was calculated to evaluate data reproducibility (RSD% < 20 % for all samples). For each batch of samples, one procedural blank (Milli-Q water), one quality control (Milli-Q water spiked with PFAS native standards), and two procedural controls with non-spiked as well as spiked drinking water were extracted simultaneously.

Internal validation of the analytical method showed a relative standard deviation of the analysis lower than 20 %. The potential sorption of PFAS to the HDPE plastic bottle wall was evaluated after 24 h and 96 h, and the data showed no PFAS sorbed to the bottle walls.

For HRMS instrument quality control, methanol injections were carried out before and after standard injections to assess any contamination in the LC system. Internal mass calibration for each analysis was performed by infusing a 2 mM sodium acetate solution in a water:methanol mixture (1:1, v:v) at the beginning of the analysis (0.1–0.5 min). The limit of quantification and recoveries are reported in Tables S1 and S3, see Ref. [14] for further details.

Results for PFBS, PFOA and PFOS, both linear and branched, were excluded from further data analysis due to the high and inconsistent detection of these compounds in the procedural blanks. Other PFAS compounds were below the limit of quantification in the blanks (Table S1).

### 2.5 Data analysis

The amount of PFAS sorbed was assessed at each point in time by calculating the difference between the initial and final PFAS concentration measured in the water phase. A complete mass balance is assumed, considering the persistence of PFAS in these experimental conditions, the shown absence of losses related to sorption onto the HDPE plastic bottle wall, and the low volatility of the compounds in general.

The sorption at each timepoint was calculated by determining the difference between the initial and final PFAS concentrations, dividing this by the initial concentration, and expressing the result as a percentage. The initial concentration was determined by summing the spiked PFAS concentration and the PFAS content present in the original drinking water, which was confirmed in triplicate through quality control involving drinking water spiked with PFAS native standards. The final concentration represents the measured PFAS concentration in the water after conducting the sorption experiment.

Sorption was observed to be rapid, occurring within the initial time interval of 1 h. Assuming equilibrium was reached after 1 h for each of the three GAC sorbents, we used the time-averaged sorption data from 1 h to 240 h to evaluate sorption performance and stability over longer time frames. To assess significant differences in the time-averaged sorption of different PFAS, we employed the non-parametric Mann-Whitney *U* test (p < 0.05) in the R statistical environment (version 4.1.2).

## 3 Results and discussion

### 3.1 Sorption of PFAS and PFAS precursors

The sorbed fraction of PFCA (C_4_–C_12_) and PFSA (C_5_–C_10_) significantly increased with chain length, as indicated by the linear regression in Fig. S1 (p < 0.05 and R ranging from 0.69 to 0.97 for all tested GAC). This suggests that hydrophobic interactions of the perfluorinated carbon chain play a dominant role in the sorption of the tested PFCA and PFSA. The results align with previous studies [29,43], where the sorption was tested at higher PFAS concentrations and GAC-to-water ratios during the experiment (considered unrealistic for environmental conditions). These studies found that a longer hydrophobic tail resulted in increased sorption on GAC. The shorter chain PFCA and PFSA exhibited relatively low sorption compared to the longer chain PFCA and PFSA, suggesting a limited role for electrostatic interactions between the anionic head group and the GAC under the experimental conditions [44].

**Fig. 2:**
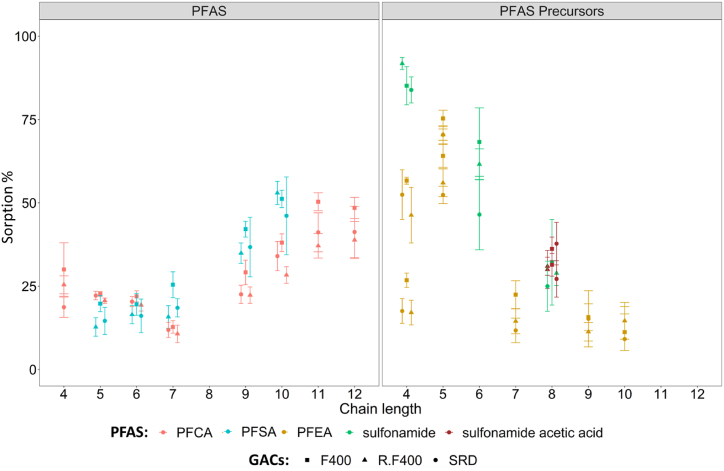
Relationship between time-averaged sorption and PFAS carbon chain length to the studied GACs (F400, R–F400, RSD) for the studied PFAS and PFAS precursors. Error bars represent the standard deviation of the time-average sorption.

PFSA consistently showed higher sorption compared to PFCA for all three sorbents (Fig. 2), except for PFSA with a C5–C6 carbon chain length. This trend can be attributed to differences in electron withdrawal between sulfonate and carboxylate groups, influencing the hydrophobic properties of perfluoroalkyl chains in both molecular and ionized forms [45]. Similarly, previous studies have also indicated stronger interactions between long-chain PFAS and sulfonated head groups on granular activated carbon (GAC) surfaces compared to PFAS with carboxylic acid heads [46,47].

No clear relation between sorption and carbon chain length could be observed for the PFAS precursors, specifically PFEA (mono- and di-ether: C_4_–C_10_), and sulfonamides (C_4_–C_8_) (Fig. 2, Table S4). However, it's important to note that the limited range of tested carbon chain lengths for the PFAS precursors (including mono-ether PFEA C_4_, C_5_, C_8_, and C_10_, di-ether C_4_ and C_5_, sulfonamides C_4_, C_6_, and C_8_, sulfonamide acetic acids C_8_) somewhat hindered the ability to reveal significant trends. Interestingly, short chain PFAS precursors generally exhibited higher sorption than short chain PFCA and PFSA, consistent with previous research findings [48,49]. Conversely, for long chain precursors, the opposite trend was observed.

The introduction of ether linkages or sulfonamide groups alters the heterogenous distribution of charge across the molecule, enabling electrostatic interactions with the activated carbon [50]. For instance, the PFEAs, specifically PF4OPeA and PF5OHxA, showed similar sorption despite their differing chain lengths, suggesting the relevance of the electrostatic interactions for these precursors (see Figs. S2 and S3). Furthermore, introducing an additional ether group to 3-6-OPFHpA (C_5_, di-ether) increased sorption compared to PF4OPeA (C_4_) and PF5OHxA (C_5_) (see Fig. S2), further supporting the relevance of the electrostatic interactions facilitated by ether groups.

While considering different functional groups, PF4OPeA and PFEESA, both C_4_ mono-ethers but differ in head functional groups (carboxylate and sulfonate respectively), are shown in Fig. S2. These PFEAs showed significantly lower sorption for carboxylate PFEAs (PF4OPeA). This difference can be attributed to the stronger electron withdrawing nature of the sulfonate group compared to the carboxylate group. Sulfonic acids are much stronger acids than carboxylic acids, withdrawing the ether electrons, thereby reducing their availability for electrostatic interactions at the ether position [45].

In the case of PFEESA, 9Cl– PF3oNS, and 11 C l-PF3oUnDS (C_4_, C_8_, and C_10_, mono-ether), longer chains (C_8_, C_10_) resulted in significantly lower sorption compared to their shorter chain C_4_ counterparts. This trend was also observed for short and long chains of di-ethers (3-6-OPFHpA, ADONA). Similarly, for FBSA, FHxSA, and FOSA (fluorinated sulfonamide with chain lengths C_4_, C_6_, and C_8_, respectively), lower sorption was observed with increasing chain length, with a significant difference between C_4_ and C_8_, Fig. S2.

Moreover, in the case of sulfonamide acetic acid C_8_ (EtFOSAA and MeFOSAA), the presence of both the sulfonamide group and carboxylic group enhanced their sorption compared to the PFCA of same chain length, like C_9_ (Fig. 2, Table S4). This can be attributed to the additional electrostatic interaction on top of hydrophobic interaction.

No significant difference in time-averaged sorption was observed between linear and branched isomers of PFHxS on all tested GAC (Table S4). However, both sulfonamide acetic acids showed significantly different sorption (p < 0.05), with higher sorption observed for branched than linear isomers. The decrease in length and increase in width of the hydrophobic tail in branched isomers, compared to linear isomers, exerts a more pronounced effect on the functional group, affecting the electrostatic interaction [51,52].

Overall, the sorption of the studied PFAS and PFAS precursors to the GAC can be explained by both hydrophobic and electrostatic interactions, with the relative contributions of each type of interactions dependent on the chemical structure.

### 3.2 Comparison of carbon sorbents

The porosity and structure of micropores and mesopores can impact the sorption of chemicals on GAC [38,53]. However, this study did not show distinct differences in sorption among the various studied classes of PFAS across the three types of studied GACs. The F400 and SRD GAC did not show significant differences and also the reactivation of F400 GAC (Table S2) did not have significant impact on sorption (Fig. 2, Fig. S2).

Nevertheless, slight variation of the sorption for each PFAS were observed over time, as summarized in Table S2 and depicted in Fig. S3. This is consistent with previous research [54,55]. The differences were negligible for F400 but more pronounced for R–F400 and SRD. This variation might be attributed to the difference in the surface area, particularly the higher volume of mesopores in the regenerated GAC (R–F400) and the newly developed GAC (SRD) compared to the virgin form (F400) (Table S1). The increase in mesopore volume on the GAC could lead to competition for sorption sites within water constituents.

In this study, the GAC was neutral to positively charged, as the water (pH = 8.6) was close to or slightly below the GAC point of zero charge (Table S2). This limited the potential for strong electrostatic interactions between the negatively charged PFAS and the GAC surface. Furthermore, the presence of organic matter (OM) and cations in water, in mg/L range [39], should be taken into consideration. OM is a mixture of different fractions (hydrophobic, hydrophilic, acids, bases and neutrals), and it is mostly expected to be negatively charged [56]. Organic matter can compete with the PFAS and PFAS precursors and also coat the GAC surface, reducing the sorbents capacity and altering its surface charge.

While Kothawala et al. showed improvement in PFAS sorption by GAC in the presence of OM, with variable responses depending on the type of OM and PFAS chain length [57], it's important to note that the complexity introduced by these various water constituents should not be underestimated. Additionally, the presence of inorganic species in the water, such as divalent cations (Ca^2+^, Mg^2+^), may compress the electrical double layer of the GAC and reduce electrostatic repulsion. This can lead to structural rearrangement and better packing of PFAS on GAC surface [[58], [59], [60]].

Considering the complexity introduced by these various water constituents, it is essential to conduct further research to gain a comprehensive understanding the influence of each of these individual water constituent on PFAS sorption on GAC surface. This may involve testing different types of organic matter or (divalent) cations at different concentrations, particularly in scenarios with low PFAS concentrations and in a real drinking water matrix to be more relevant to the drinking water production.

### 3.3 Implications for water production

This study found relatively low removal percentages for PFAS (>50 %), except for the short chain PFAS precursors, at environmentally relevant concentrations and using a water to GAC ratio (100 m^3^ for one kg of GAC). This ratio is in the same range as commonly applied GAC to water ratios at the end of a GAC bed lifetime [40,41], and application of PAC in drinking water treatment [42]. The progressively stringent advised safety levels for drinking water impose significant pressure on water producers to ensure the removal of PFAS using GAC below the advised safe threshold. This indicates that (carbon) sorbents, process conditions, management and maintenance of drinking water production to remove PFAS from drinking water requires improvement to meet potential future thresholds. Improvements to sorbents such as increased fluorophilicity, hydrophobicity, and affinity for chemicals carrying a negative charge have been suggested [61]. Alternatively, reverse-osmosis membrane-based treatments demonstrated efficient removal of low PFAS concentrations from source water, and may currently be the most viable alternative to carbon sorbents [5,14].

## 4 Conclusion

This study investigated the sorption behavior of PFAS, including PFCA, PFSA, and three classes of PFAS precursors, on GAC at environmentally relevant concentrations (∼5 ng/L), with a focus on their implications for drinking water production. Our findings reveal that the sorption of PFAS onto GAC at these realistic concentrations, taking into account GAC dosing relevant to drinking water treatment, was relatively low for all studied PFAS and PFAS precursors, except for the short-chain PFAS precursors.

The sorption of PFAS was found to be influenced by both the carbon chain length and functional groups present, with a dominant role played by both electrostatic and hydrophobic interactions. Notably, while no clear linear relationship between sorption and chain length emerged for PFAS precursors, the introduction of ether linkages or sulfonamide groups led to significant alterations in their sorption behavior. Interestingly, the presence of branched isomers had varying effects, with no significant impact observed for PFSA, but significantly higher sorption for sulfonamide acetic acids.

Furthermore, the characteristics of the three forms of GAC tested did not significantly influence the sorption of PFAS. However, it's worth noting that the complexities introduced by various water constituents, such as organic matter and divalent cations, could affect sorption mechanisms and require further investigated.

This study highlights the challenges associated with achieving efficient PFAS removal in drinking water production. Future research should continue to explore the influence of different water constituents on PFAS sorption on GAC and/or new types of sorbents as well as other treatment concepts such as reverse-osmosis. This should be studied particularly under realistic environmental PFAS concentrations and treatment conditions, to both enhance our understanding and improve drinking water treatment to meet current and future challenges.

## Data availability

Data will be made available on request.

## CRediT authorship contribution statement

**Mohammad Sadia:** Writing – original draft, Visualization, Methodology, Data curation, Conceptualization. **Lola Beltrán Beut:** Investigation, Formal analysis. **Marko Pranić:** Writing – review & editing, Methodology. **Annemarie P.van Wezel:** Writing – review & editing, Project administration, Methodology, Funding acquisition, Conceptualization. **Thomas L.ter Laak:** Writing – review & editing, Validation, Methodology, Conceptualization.

## Declaration of competing interest

The authors declare that they have no known competing financial interests or personal relationships that could have appeared to influence the work reported in this paper.
