# Supplementary material for: Sorption of per- and poly-fluoroalkyl substances and their precursors on activated carbon under realistic drinking water conditions: Insights into sorbent variability and PFAS structural effects
Source: Heliyon. 2024 Jan 26;10(3):e25130. doi: 10.1016/j.heliyon.2024.e25130 (PMC10839585; doi:10.1016/j.heliyon.2024.e25130)
Supplement: Multimedia component 1 [file mmc1.docx]

Supplementary information

**Sorption of Per- and Poly-fluoroalkyl Substances and their Precursors on Activated Carbon under Realistic Drinking Water Conditions: Insights into Sorbent Variability and PFAS Structural Effects**

Mohammad Sadia^a^, Lola Beltrán Beut^a^, Marko Pranić^b^, Annemarie P. van Wezel^a^, Thomas L. ter Laak^a,c^

^a^Institute for Biodiversity and Ecosystem Dynamics, University of Amsterdam, Science Park 904, 1098XH Amsterdam, The Netherlands.

^b^Environmental Technology, Wageningen University, Bornse Weilanden 9, 6708WG, Wageningen, the Netherlands

^c^KWR Water Research Institute, Groningenhaven 7, 3430BB Nieuwegein, The Netherlands.

## **Standards and materials**

Native and isotopic mass labelled standards (Table S1) were purchased from Wellington Laboratories (Guelph, Canada), excluding *n-*deuteriomethylperfluoro-1-n-octanesulfonamidoacetic acid-d_3_ (N-MeFOSAA-d_3_, >99%) and n-ethylperfluoro-1-n-octanesulfonamidoacetic acid-d_5_ (N-EtFOSAA-d_5_, >99%) which were purchased from Chiron (Trondheim, Norway), trifluoroacetic acid (TFA, >99%) and perfluoropropanoic acid (PFPrA, >97%) from Sigma-Aldrich (Darmstadt, Germany), perfluoroethane sulfonic acid (PFEtS, >98%) from Kanto Chemical (Japan), and n-methylperfluorobutanesulfonamide (MeFBSA, >97%) from Apollo scientific (Manchester, United Kingdom). The LC-MS grade methanol and acetonitrile were purchased from Biosolve Chimie (Dieuze, France), the ammonium acetate (≥99%) and glacial acetic acid (≥99%) from Sigma-Aldrich and the ammonia solution (25%, analytical reagent grade) was purchased from Fisher Scientific (Massachusetts, United States).

## **Chemical analysis**

Extraction and quantification procedures were reported in detail elsewhere [1]. Briefly, samples were adjusted to pH 4 using acetic acid. Mass-labeled extraction standards (10 µL; 0.1-0.2 ng/µL in methanol) were spiked. The samples were then loaded onto weak anion exchange Waters Oasis® WAX-SPE cartridges (3 mL, 60 mg, 30 μm; Waters Corporation Milford, USA) that were preconditioned with 3 mL of 0.1% ammonium hydroxide in methanol, followed by 3 mL of methanol, and finally 3 mL Milli-Q water. After loading the samples, the cartridges were washed with 3 mL ammonium acetate buffer solution at pH 4, and dried for ∼1h under vacuum. Elution was performed with 3 mL of 0.1% ammonium hydroxide in methanol. The extracts were evaporated under high-purity nitrogen to 65 µL, followed by the addition of 175 µL of 0.05% acetic acid in water and 10 µL of mass-labelled injection standard solution (IS; 0.1 ng/µL). The 250 µL extracts were vortex-mixed, centrifuged (5 min at 4000 rpm), and then transferred to LC vial for further chemical analysis.

Quantification was carried out using a Nexera UHPLC system (Shimadzu, Kyoto, Japan) coupled to a Bruker MaXis 4 G high-resolution q-TOF-HRMS, equipped with an Ion Booster Electro Spray Ionization (IB-ESI) source. Aliquots of 5 µL were injected into an Acquity UPLC CSH C18 column (130 Å, 2.1 x 150 mm, 1.7 µm) for analysis. The mobile phase consisted of 0.05% acetic acid in water (A) and 0.05% acetic acid in acetonitrile (B). Identification of the targeted PFAS (Table S2) was performed using the accurate mass, retention time, and fragment ions [1]. Quantification was conducted based on the relative response factor (RF) using corresponding extraction standard. A total of 10 points external calibration curve consisting of a series of concentrations ranging from 50 to 6000 pg/mL (correlation coefficients >0.99), was used to quantify the target analyte concentration. Branched isomers for PFHxS, EtFOSAA, and MeFOSAA were quantified using the branched isomer standard.

# **Particle porosity measurements**

The granular activated carbon (GAC) skeletal (material) and particle (apparent) densities were measured with the pycnometer with water as pycnometer liquid, as described in detail in [2]. With this method only the pores that are accessible to water are considered in density measurements. Around 3 g of dry GAC was weighted and placed in water which was then boiled to ensure complete wetting of GAC. The wet GAC was removed from boiling water and rolled on cellulose tissues to remove the excess water i.e., water that is present on the external surface of the particles. In this way, GAC particles whose pores are filled with water are obtained, and their mass was measured in a pre-weighted pycnometer. After that, the pycnometer was filled with water and weighted again. With the obtained masses, and known pycnometer volume, the material and particle density were calculated according to equations reported in Sontheimer et al. In the end, particle porosity was calculated from the following equation:

$\varepsilon_{p}=1-\frac{\rho_{p}}{\rho_{m}}$ (S7)

where $\varepsilon_{p}$ (dimensionless) is particle porosity, $\rho_{P}$ (g/L) is particle density and $\rho_{m}$ (g/L).is material density

# **pH drift method**

The pH drift method was used to determine the pH point (pH_PZC_) at which the surface of carbon contains equal amounts of positively and negatively charged functional groups, i.e., surface is globally neutral. With this method, activated carbons were equilibrated with NaCl solutions of different starting pH. Because of the protonation and deprotonation of activated carbon functional groups, the pH of the solution will change, and, in equilibrium it will be constant. Experiments were performed in glass bottles containing 0.5 L of 0.01 M NaCl. The starting pH was adjusted with the addition of HCl or NaOH to be between 2-11. After adjustment, around 1.0 g of GAC was added, and the system was stirred on a magnetic stirrer for 48 h. After 48 h system was in equilibrium, and longer experiments showed no significant change in the final pH. During each step of the experiment, NaCl solution was sparged with N_2_ to remove the impact of CO_2_ on the solution pH. The pH_PZC_ was obtained by plotting initial vs final pH, and the resulting graph is shown in Figure S.2. The point where initial vs final pH curve crosses y = x line is pH_PZC_. For the pH bellow pH_PZC_ surface is positively charged, and negatively charged beyond.

| 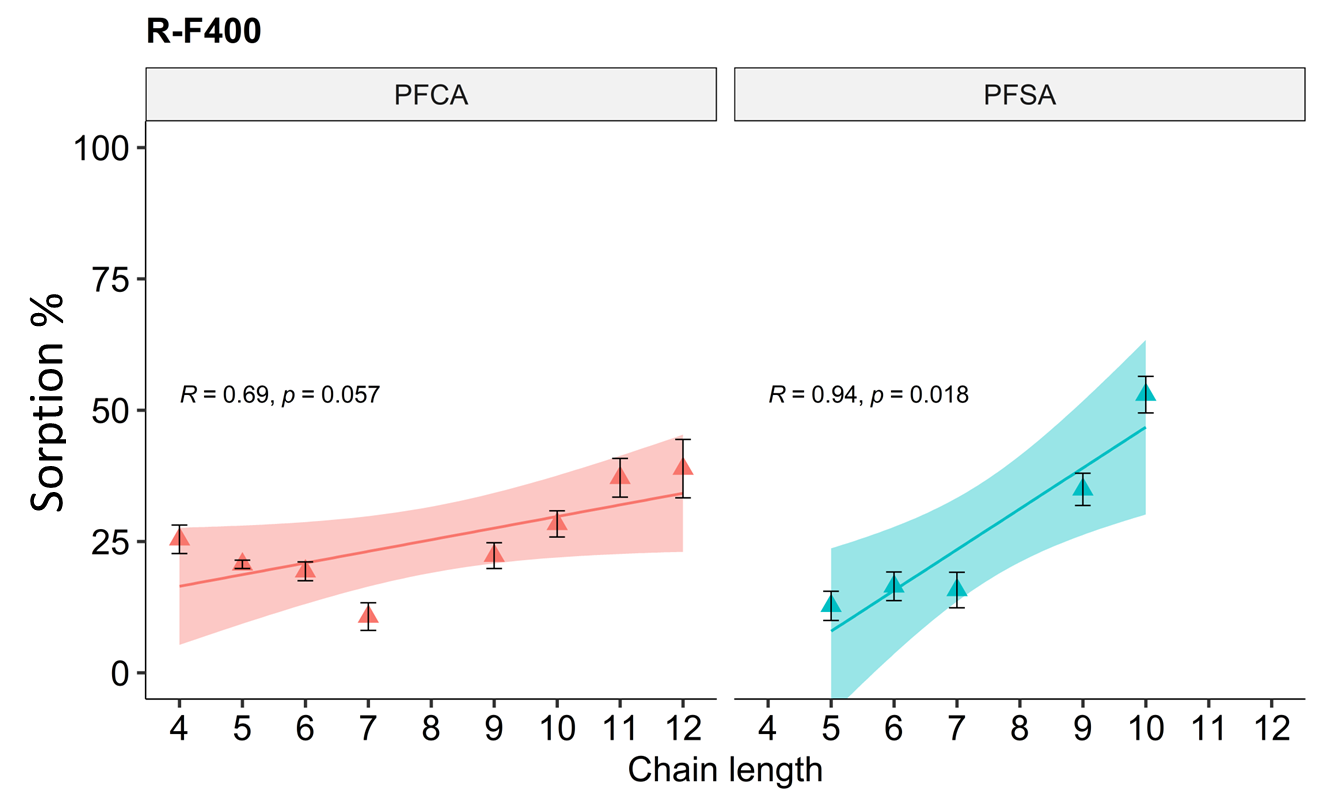 |
| --- |
| 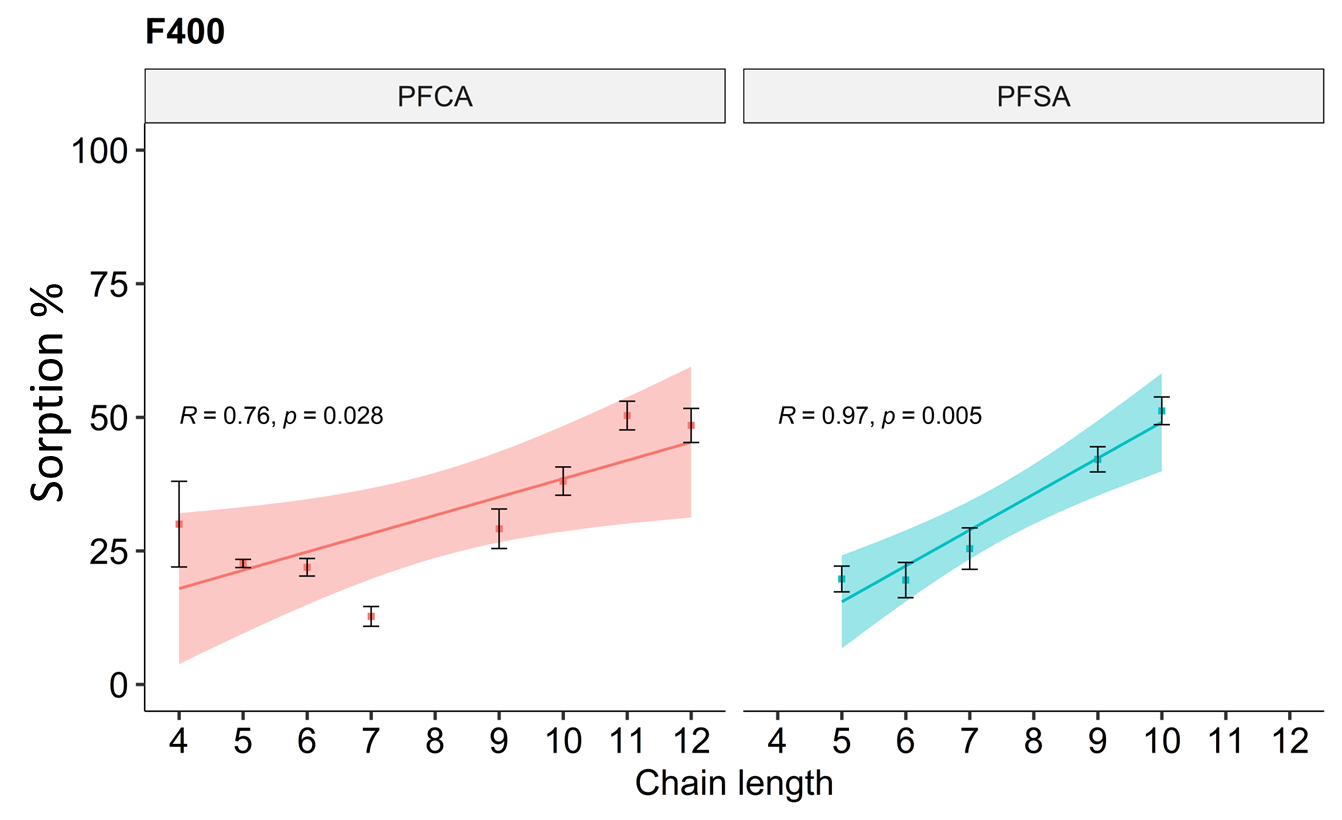 |
| 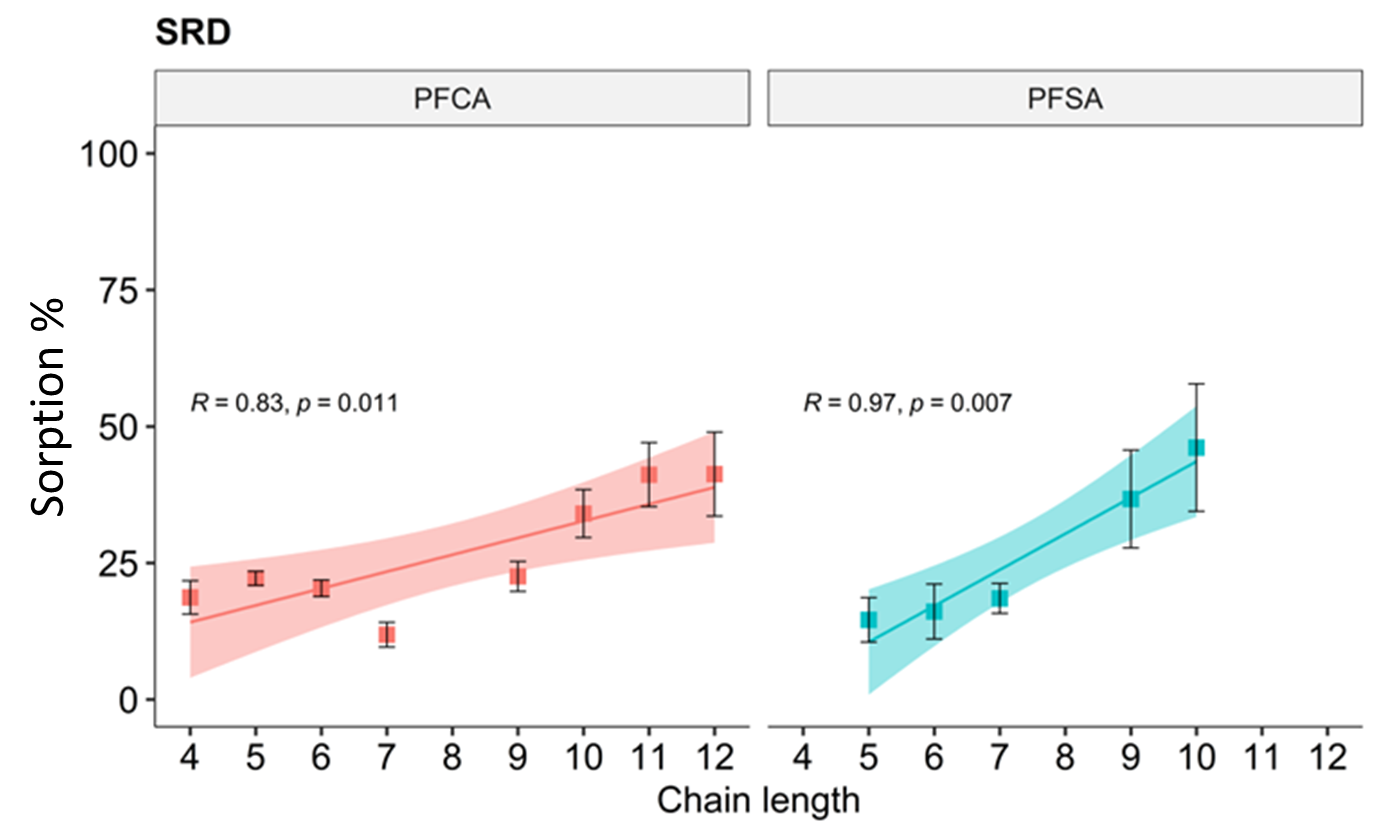 |
| **Figure S1**. Relationship between the PFAS carbon chain length and the time-averaged sorption for the studied PFAS to studied GAC. Error bars represent the standard deviation of the time-average sorption data and the shaded area represent the 95% confidence limits. |

| 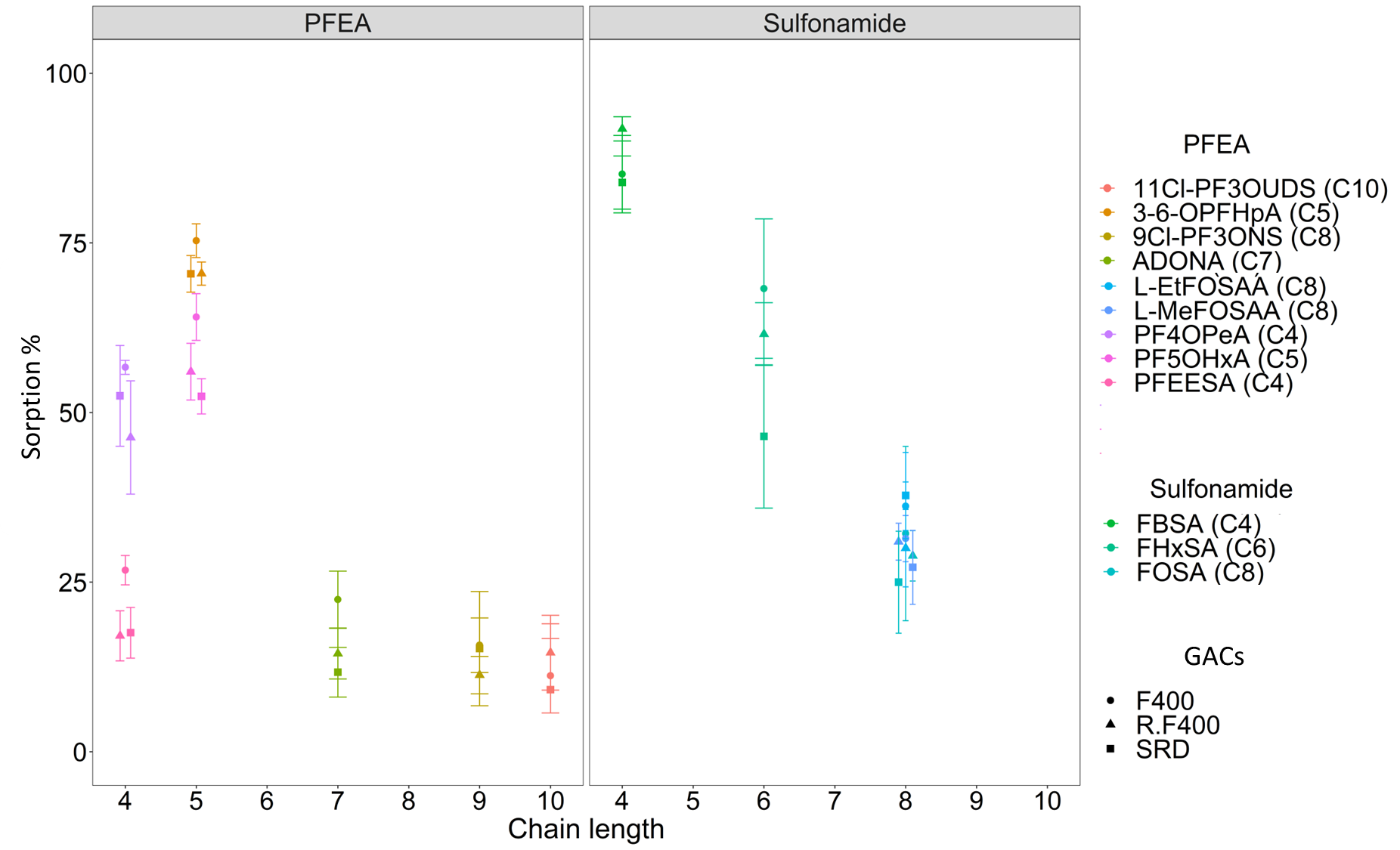 |
| --- |
| **Figure S2**. Relationship between the PFAS precursors (per-/poly-fluoroalkyl ether acids (PFEA), fluorinated sulfonamides) carbon chain length and the time-average sorption for the studied PFAS to studied GAC. Error bars represent the standard deviation of the time-average sorption data. |

| 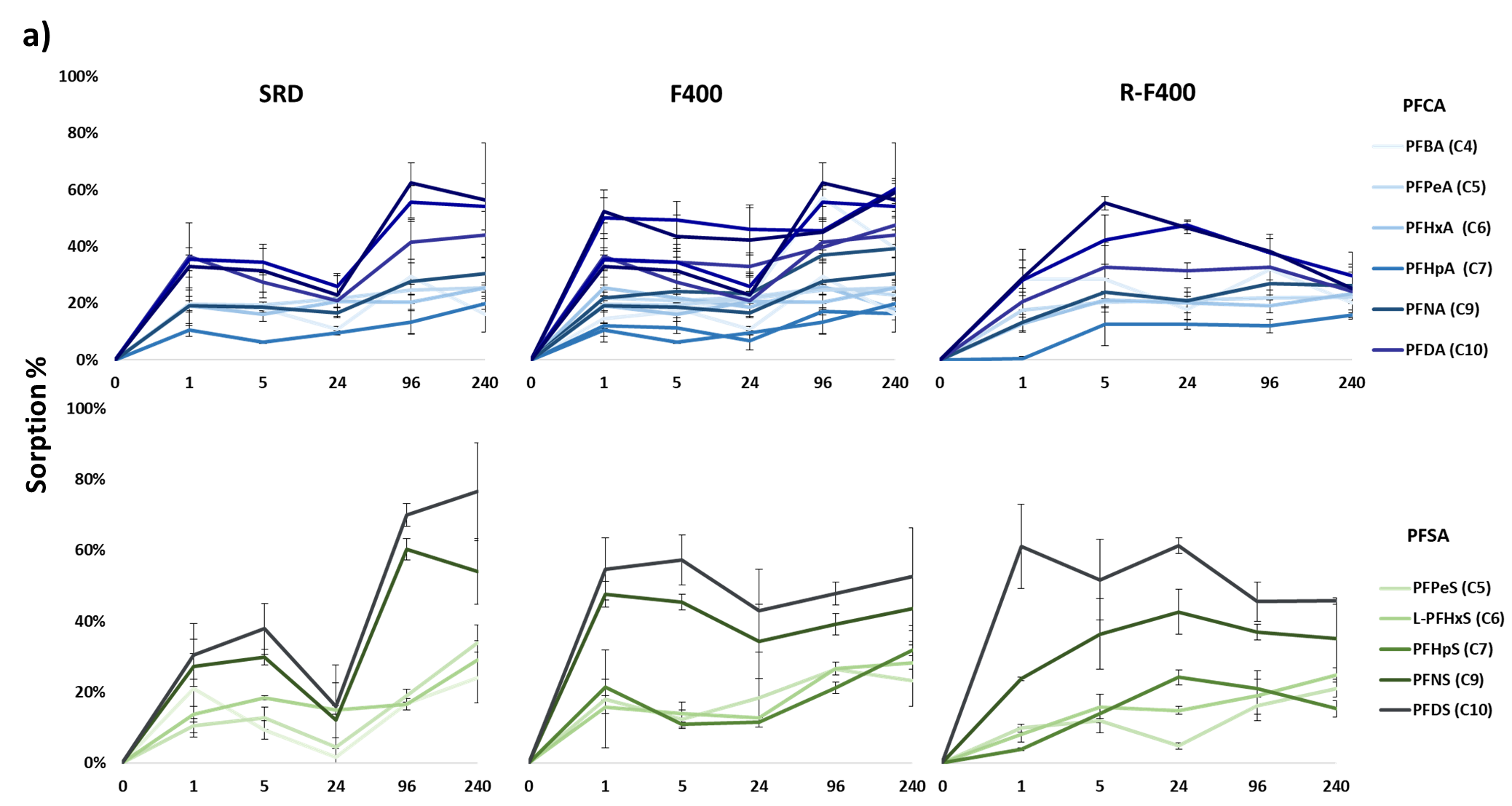 |
| --- |
| 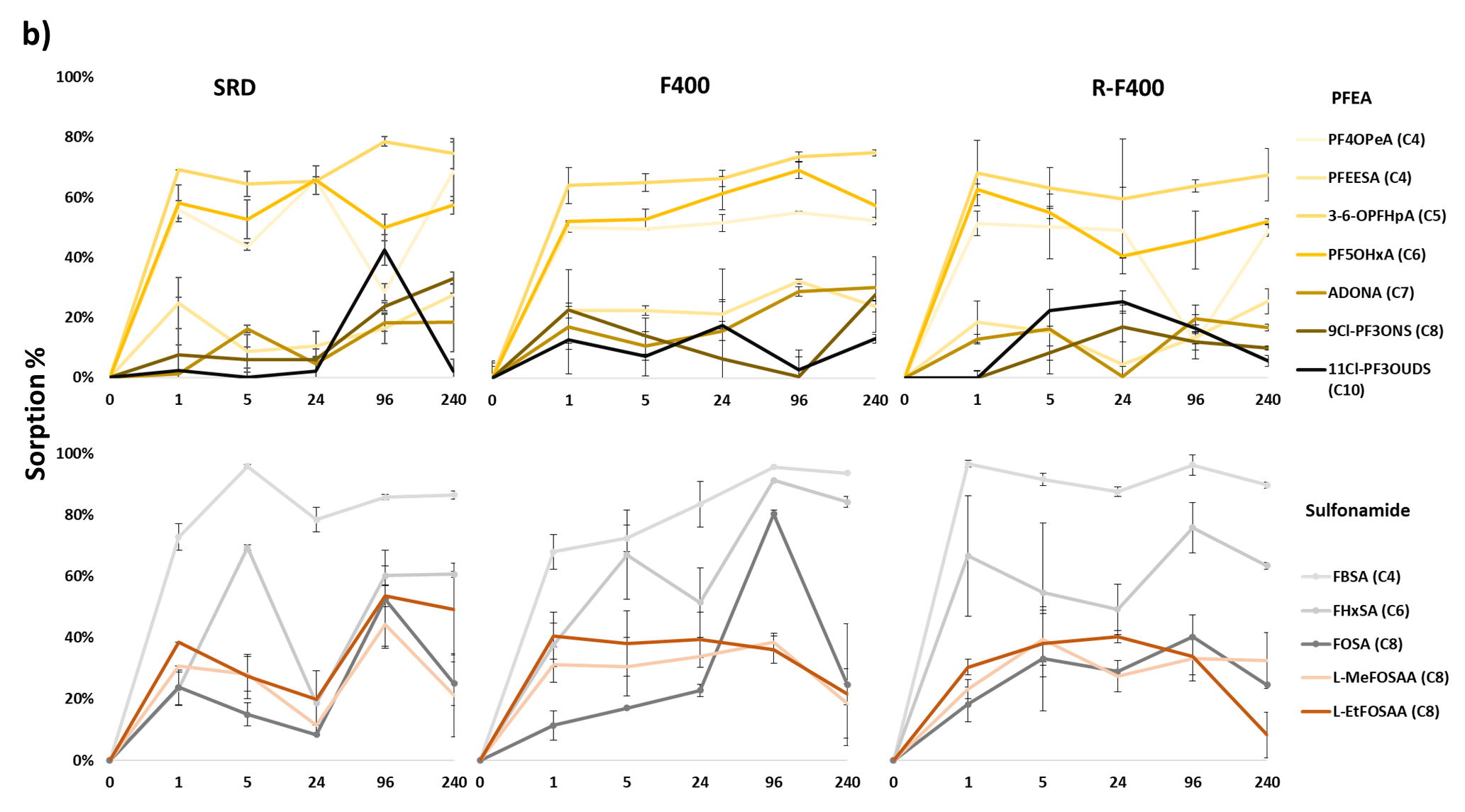 |
| 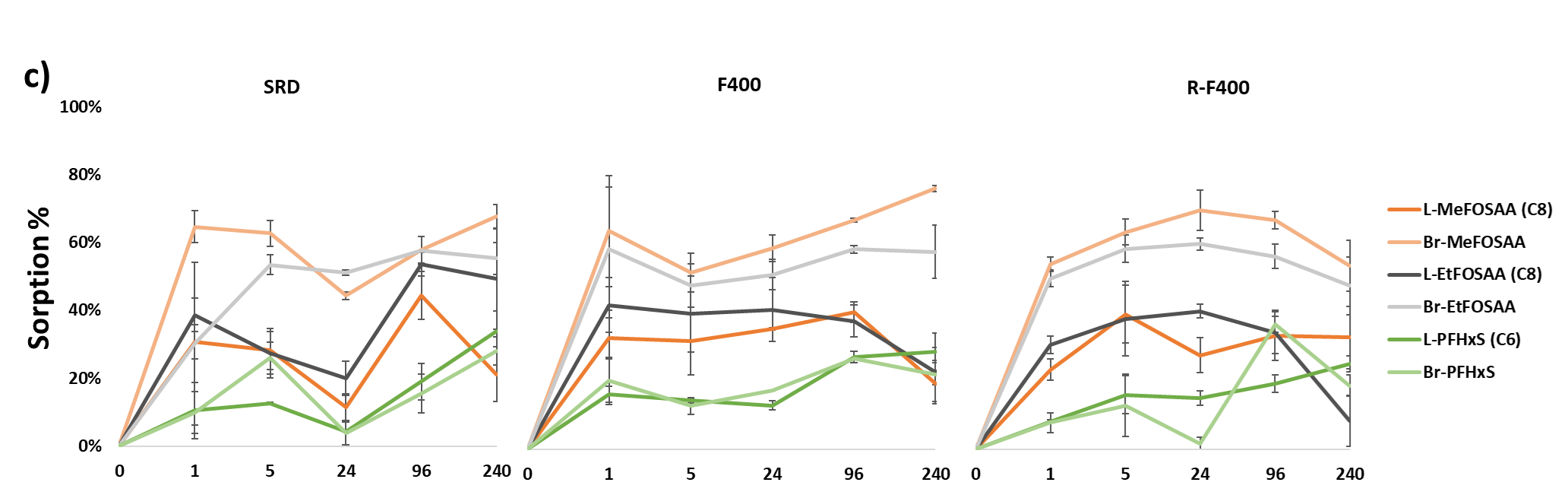 |
| **Figure S3.** Percentage sorbed over exposure time (h) for the studied sorbents SRD, F400, and R-F400. PFAS are organized in classes: a) perfluoro-carboxylic acids (PFCA) and perfluoro-sulfonic acid (PFSA); b) fluorinated sulfonamides, per-/poly-fluoroalkyl ether acids (PFEA); c) studied branched (Br) and linear (L) isomer of both PFAS and PFAS precursors. The error bars represent the standard deviation of triplicate samples. |

**Table S1.** The limit of quantification (LOQ) of all the targeted PFAS, PFAS classes.

| PFAS class | | Compound | Acronym and chain length | Mass-labelled | LOQ (pg/L) |
| --- | --- | --- | --- | --- | --- |
| perfluorocarboxylic acids (PFCA) | | Perfluorobutyric acid | PFBA (C4) | M4PFBA | 50 |
|  |  | Perfluoropentanoic acid | PFPeA (C5) | M5PFPeA | 50 |
|  |  | Perfluorohexanoic acid | PFHxA (C6) | M5PFHxA | 50 |
|  |  | Perfluoroheptanoic acid | PFHpA (C7) | M4PFHpA | 50 |
|  |  | Perfluorooctanoic acid | PFOA (C8) | M8PFOA | - |
|  |  | Perfluorononanoic acid | PFNA (C9) | M9PFNA | 50 |
|  |  | Perfluorodecanoic acid | PFDA (C10) | M6PFDA | 50 |
|  |  | Perfluoroundecanoic acid | PFUdA (C11) | M7PFUdA | 50 |
|  |  | Perfluorododecanoic acid | PFDoA (C12) | MPFDoA | 50 |
| perfluorosulfonic acids (PFSA) | | Potassium perfluoro-1-butanesulfonate | PFBS (C4) | M3PFBS | 44 |
|  |  | Sodium perfluoro-1-pentanesulfonate | PFPeS (C5) | M3PFBS | 47 |
|  |  | Potassium perfluorohexanesulfonate | L-PFHxS (C6) | M3PFHxS | 37 |
|  |  |  | Br-PFHxS (C6) | M3PFHxS | 9 |
|  |  | Sodium perfluoro-1-heptanesulfonate | PFHpS (C7) | M3PFHxS | 48 |
|  |  | Potassium perfluoro-octanesulfonate | PFOS (C8) | M8PFOS | - |
|  |  | Sodium perfluoro-1-nonanesulfonate | PFNS (C9) | M8PFOS | 48 |
|  |  | Sodium perfluoro-1-decanesulfonate | PFDS (C10) | M8PFOS | 48 |
| Precursors | *Sulfonamide* | Perfluorobutylsulfonamide | FBSA (C4) | M3PFBS | 50 |
|  |  | Perfluorohexanesulfonamide | FHxSA (C6) | M3PFHxS | 50 |
|  |  | Perfluorooctanesulfonamide | FOSA (C8) | M8PFOS | 50 |
|  | *sulfonamide acetic acids* | N-methylperfluorooctane sulfonamidoacetic acid | L-MeFOSAA (C8) | d5-N-EtFOSAA | 38 |
|  |  |  | Br-MeFOSAA (C8) | d5-N-EtFOSAA | 24 |
|  |  | N-ethylperfluorooctane sulfonamidoacetic acid | L-EtFOSAA (C8) | d5-N-EtFOSAA | 39 |
|  |  |  | Br-EtFOSAA (C8) | d5-N-EtFOSAA | 12 |
|  | *per-/poly-fluoroalkyl ether acids (PFEA)* | Sodium dodecafluoro-3H-4,8-dioxanonanoate | ADONA (C7) | M8PFOA | 47 |
|  |  | Potassium 9-chlorohexadecafluoro-3-oxanonane-1-sulfonate | 9Cl-PF3ONS (C8) | M8PFOA | 47 |
|  |  | Potassium 11-chloroeicosafluoro-3-oxaundecane-1-sulfonate | 11Cl-PF3OUDS (C10) | M8PFOA | 47 |
|  |  | Perfluoro-4-oxapentanoic acid | PF4OPeA (C4) | M4PFBA | 50 |
|  |  | Perfluro-5-oxahexanoic acid | PF5OHxA (C5) | M3PFHxS | 50 |
|  |  | Perfluoro-3,6-dioxaheptanoic acid | 3-6-OPFHpA (C5) | M8PFOA | 50 |
|  |  | Potassium perfluoro(2-ethoxyethane)sulfonate | PFEESA (C4) | M3PFHxS | 45 |

**Table S2.** Properties of the three types of GAC

|  | Density  (Kg/L) | Micropore volume  (cm^3^/g) | Mesopore volume  (cm^3^/g) | Particle porosity  (%) | pH point of zero charge |
| --- | --- | --- | --- | --- | --- |
| F400 | 0.51 | 0.32 | 0.12 | 50 | 9.4 |
| F400-R | 0.42 | 0.26 | 0.26 | 54 | 8.8 |
| SRD | 0.46 | 0.36 | 0.23 | 58 | 8.4 |

**Table S3.** Recoveries of the extraction and injection standard.

|  | Compound | Acronym | Recovery (SD) |
| --- | --- | --- | --- |
| Extraction standard | Perfluoro-n-[^13^C4]butanoic acid | M4PFBA | 102% (14.7) |
|  | Perfluoro-n-[^13^C5]pentanoic acid | M5PFPeA | 86% (12.4) |
|  | Perfluoro-n-[1,2,3,4,6-^13^C5]hexanoic acid | M5PFHxA | 78% (21.2) |
|  | Perfluoro-n-[1,2,3,4-^13^C4]heptanoic acid | M4PFHpA | 81% (19.2) |
|  | Perfluoro-n-[^13^C8]octanoic acid | M8PFOA | 83% (16.9) |
|  | Perfluoro-n-[^13^C9]nonanoic acid | M9PFNA | 90% (21.0) |
|  | Perfluoro-n-[1,2,3,4,5,6-^13^C6]decanoic acid | M6PFDA | 81% (24.7) |
|  | Perfluoro-n-[1,2,3,4,5,6,7-^13^C7]undecanoic acid | M7PFUdA | 102% (33.3) |
|  | Perfluoro-n-[1,2-^13^C]dodecanoic acid | MPFDoA | 162% (22.1) |
|  | Sodium perfluoro-1-[2,3,4-^13^C3]butanesulfonate | M3PFBS | 65% (22.7) |
|  | Sodium perfluoro-1-[1,2,3-^13^C3]hexanesulfonate | M3PFHxS | 81% (29.7) |
|  | Sodium perfluoro-1-[^13^C8]octanesulfonate | M8PFOS | 80% (25.9) |
| Injection standard | Perfluoro-n-[2,3,4-^13^C3]butanoic acid | M3PFBA |  |
|  | Perfluoro-n-(1,2-^13^C2]octanoic acid | M2PFOA |  |
|  | Sodium perfluoro-1-[1,2,3,4-^13^C4]octanesulfonate | M4PFOS |  |

**Table S4.** Percentage sorbed of individual PFAS on the three studied GAC for individual exposure times(1, 5, 24, 96, 240 h) and the time-averaged sorption of 1h to 240h (Av). Data represents triplicate samples, with standard deviation indicated in parentheses.

| PFAS classes | | *Compounds* | *F400* | | | | | | *R-F400* | | | | | |
| --- | --- | --- | --- | --- | --- | --- | --- | --- | --- | --- | --- | --- | --- | --- |
|  |  |  | *1* | *5* | *24* | *96* | *240* | ***Av*** | *1* | *5* | *24* | *96* | *240* | ***Av*** |
| perfluorocarboxylic acids (PFCA) | | PFBA (C4) | 14,5% (8) | 17,2% (2) | 22% (2) | 57,1% (3) | 39,2% (11) | **30% (16)** | 28,4% (11) | 28,6% (12) | 17,8% (3) | 32% (11) | 20,3% (5) | **25,42% (5)** |
|  |  | PFPeA (C5) | 21,9% (2) | 20,9% (1) | 22% (1) | 25,4% (1) | 23% (2) | **22,64% (2)** | 17,7% (1) | 20,3% (3) | 21,3% (3) | 21,9% (1) | 22% (3) | **20,64% (2)** |
|  |  | PFHxA (C6) | 25,3% (7) | 22% (1) | 18,6% (3) | 25,9% (1) | 17,9% (4) | **21,94% (3)** | 12,8% (3) | 21,2% (2) | 20,1% (2) | 19% (1) | 23,5% (2) | **19,32% (4)** |
|  |  | PFHpA (C7) | 12,1% (1) | 11,4% (2) | 6,8% (3) | 17,2% (1) | 16,2% (6) | **12,74% (4)** | 0,6% (7) | 12,5% (2) | 12,5% (2) | 12% (2) | 15,9% (2) | **10,7% (5)** |
|  |  | PFNA (C9) | 21,9% (5) | 24,2% (1) | 23,4% (3) | 36,9% (2) | 39,3% (11) | **29,14% (7)** | 13,4% (5) | 23,9% (4) | 21% (4) | 27% (6) | 26,2% (0) | **22,3% (5)** |
|  |  | PFDA (C10) | 35,2% (3) | 34,6% (4) | 33,1% (4) | 39,8% (2) | 47,6% (12) | **38,06% (5)** | 20,6% (9) | 32,6% (3) | 31,5% (5) | 32,8% (7) | 24,2% (12) | **28,34% (5)** |
|  |  | PFUdA (C11) | 50% (7) | 49,3% (7) | 46,2% (9) | 45,6% (4) | 60,5% (3) | **50,32% (5)** | 28,2% (9) | 42,4% (2) | 47,6% (5) | 37,6% (8) | 29,8% (4) | **37,12% (7)** |
|  |  | PFDoA (C12) | 52,3% (8) | 43,7% (7) | 42,2% (11) | 45% (5) | 59,2% (4) | **48,48% (6)** | 28,9% (2) | 55,3% (2) | 46,7% (6) | 38,2% (9) | 25,2% (4) | **38,86% (11)** |
| perfluorosulfonic acids (PFSA) | | PFPeS (C5) | 18,1% (2) | 12,4% (3) | 18,5% (0) | 26,5% (2) | 23,2% (5) | **19,74% (5)** | 9,8% (0) | 11,9% (1) | 4,8% (7) | 16,1% (2) | 21,1% (14) | **12,74% (6)** |
|  |  | L-PFHxS (C6) | 15,9% (2) | 14,1% (1) | 12,7% (1) | 26,7% (2) | 28,3% (5) | **19,54% (7)** | 8% (5) | 15,8% (2) | 14,8% (3) | 19% (2) | 24,7% (14) | **16,46% (5)** |
|  |  | Br-PFHxS | 19,8% (7) | 12,5% (2) | 17% (0) | 26,3% (0) | 21,7% (8) | **19,46% (5)** | 7,7% (9) | 12,7% (3) | 1,6% (2) | 36,2% (3) | 18,4% (6) | **15,32% (12)** |
|  |  | PFHpS (C7) | 21,4% (4) | 10,9% (2) | 11,5% (11) | 21,3% (3) | 32% (9) | **19,42% (8)** | 4,4% (10) | 14% (6) | 24,1% (2) | 21% (10) | 15,3% (1) | **15,76% (7)** |
|  |  | PFNS (C9) | 47,7% (9) | 45,5% (7) | 34,5% (12) | 39,2% (3) | 43,7% (14) | **42,12% (5)** | 23,8% (11) | 36,3% (2) | 42,5% (6) | 36,9% (1) | 35,1% (6) | **34,92% (6)** |
|  |  | PFDS (C10) | 54,9% (8) | 57,5% (8) | 43% (9) | 47,9% (5) | 52,7% (2) | **51,2% (5)** | 60,9% (9) | 51,6% (5) | 61,2% (2) | 45,5% (3) | 45,6% (15) | **52,96% (7)** |
| Precursors | Sulfonamide | FBSA | 70% (6) | 74,6% (4) | 86% (8) | 98,6% (0) | 96,5% (0) | **85,14% (11)** | 96,1% (1) | 91% (1) | 87% (3) | 95,7% (0) | 89,2% (4) | **91,8% (4)** |
|  |  | FHxSA | 38,7% (7) | 69% (15) | 52,9% (12) | 94% (0) | 86,7% (2) | **68,26% (21)** | 66,2% (23) | 54,3% (8) | 48,9% (8) | 75,4% (1) | 63% (5) | **61,56% (9)** |
|  |  | FOSA | 11,8% (5) | 17,5% (0) | 23,4% (2) | 82,7% (1) | 25,4% (20) | **32,16% (26)** | 18,2% (17) | 33% (0) | 28,8% (7) | 40% (0) | 24,4% (6) | **28,88% (7)** |
|  | sulfonamide acetic acids | L-MeFOSAA | 32,1% (6) | 31,4% (10) | 34,8% (0) | 39,7% (3) | 19,1% (12) | **31,42% (7)** | 23,1% (8) | 39,1% (5) | 27,2% (7) | 33% (9) | 32,4% (7) | **30,96% (5)** |
|  |  | Br-MeFOSAA | 63,2% (16) | 51,2% (6) | 58,2% (4) | 66,4% (1) | 75,6% (1) | **62,92% (8)** | 53,7% (4) | 63% (6) | 69,3% (3) | 66,3% (7) | 53,2% (5) | **61,1% (7)** |
|  |  | L-EtFOSAA | 41,8% (8) | 39,2% (11) | 40,5% (9) | 37,2% (5) | 22,2% (4) | **36,18% (7)** | 30,2% (11) | 37,9% (2) | 40% (6) | 33,7% (7) | 8,2% (8) | **30% (11)** |
|  |  | Br-EtFOSAA | 58,1% (18) | 47,4% (6) | 50,6% (4) | 58% (1) | 57,2% (8) | **54,26% (4)** | 49,5% (4) | 58,1% (2) | 59,5% (4) | 55,8% (8) | 47,4% (24) | **54,06% (5)** |
|  | *per-/poly-fluoroalkyl ether acids (PFEA)* | ADONA | 18,6% (6) | 11,8% (5) | 17,2% (3) | 31,5% (2) | 33,1% (5) | **22,44% (8)** | 14,1% (12) | 17,9% (16) | 0,3% (1) | 21,6% (2) | 18,4% (25) | **14,46% (7)** |
|  |  | 9Cl-PF3ONS | 25% (17) | 15,4% (7) | 7% (12) | 0,7% (10) | 30,4% (5) | **15,7% (11)** | 4,9% (5) | 9,1% (16) | 18,6% (6) | 13,2% (3) | 10,8% (49) | **11,32% (5)** |
|  |  | 11Cl-PF3OUDS | 13,9% (2) | 8% (7) | 19,1% (12) | 3,2% (0) | 14,4% (5) | **11,72% (6)** | 2% (7) | 24,5% (22) | 27,6% (3) | 17,9% (5) | 6,2% (0) | **15,64% (10)** |
|  |  | PF4OPeA | 54,9% (2) | 54,4% (0) | 56,5% (3) | 60,3% (0) | 57,2% (1) | **56,66% (2)** | 56,2% (2) | 55% (1) | 53,7% (11) | 13% (1) | 53,7% (3) | **46,32% (17)** |
|  |  | PF5OHxA | 57% (0) | 57,8% (4) | 67,2% (6) | 75,7% (3) | 62,7% (6) | **64,08% (7)** | 68,7% (1) | 60,1% (4) | 44,3% (5) | 50,1% (1) | 56,9% (6) | **56,02% (8)** |
|  |  | 3-6-OPFHpA | 70,1% (7) | 71,2% (3) | 72,7% (3) | 80,6% (2) | 82% (1) | **75,32% (5)** | 74,5% (3) | 69% (6) | 65,2% (3) | 69,8% (1) | 73,9% (8) | **70,48% (3)** |
|  |  | PFEESA | 24,6% (3) | 24,6% (2) | 23,3% (5) | 35,2% (1) | 26,1% (2) | **26,76% (4)** | 20,4% (8) | 16,9% (4) | 5,3% (1) | 14,9% (2) | 27,9% (9) | **17,08% (7)** |

| *PFAS classes* | | *Compounds* | *SRD* | | | | | |
| --- | --- | --- | --- | --- | --- | --- | --- | --- |
|  |  |  | *1* | *5* | *24* | *96* | *240* | ***Av*** |
| *perfluorocarboxylic acids (PFCA)* | | PFBA (C4) | 18,8% (7) | 18,2% (1) | 10,8% (1) | 29,5% (20) | 16,1% (6) | **18,68% (6)** |
|  |  | PFPeA (C5) | 19,7% (0) | 19,3% (2) | 21,6% (0) | 24,8% (2) | 25,5% (1) | **22,18% (3)** |
|  |  | PFHxA (C6) | 19,1% (0) | 16,1% (3) | 20,7% (0) | 20,4% (3) | 25,5% (2) | **20,36% (3)** |
|  |  | PFHpA (C7) | 10,5% (1) | 6,3% (4) | 9,5% (1) | 13,2% (4) | 19,8% (2) | **11,86% (5)** |
|  |  | PFNA (C9) | 19,2% (2) | 18,6% (8) | 16,7% (2) | 27,7% (8) | 30,5% (10) | **22,54% (5)** |
|  |  | PFDA (C10) | 36,5% (2) | 27,3% (7) | 20,8% (2) | 41,5% (7) | 44,1% (8) | **34,04% (9)** |
|  |  | PFUdA (C11) | 35,5% (4) | 34,6% (6) | 25,9% (4) | 55,7% (6) | 54,1% (8) | **41,16% (12)** |
|  |  | PFDoA (C12) | 33,1% (8) | 31,4% (7) | 22,9% (8) | 62,5% (7) | 56,4% (20) | **41,26% (15)** |
| *perfluorosulfonic acids (PFSA)* | | PFPeS (C5) | 21,1% (6) | 9,3% (6) | 1,7% (6) | 16,7% (6) | 24,1% (0) | **14,58% (8)** |
|  |  | L-PFHxS (C6) | 10,5% (0) | 12,7% (5) | 4,4% (0) | 19% (5) | 33,9% (6) | **16,1% (10)** |
|  |  | Br-PFHxS | 9,9% (2) | 25,9% (6) | 3,8% (2) | 15,5% (6) | 28,1% (3) | **16,64% (9)** |
|  |  | PFHpS (C7) | 13,7% (3) | 18,3% (7) | 14,9% (3) | 16,6% (7) | 29% (7) | **18,5% (5)** |
|  |  | PFNS (C9) | 27,2% (9) | 29,8% (6) | 12,2% (9) | 60,3% (6) | 54,1% (12) | **36,72% (18)** |
|  |  | PFDS (C10) | 30,4% (5) | 37,8% (4) | 15,9% (5) | 70% (4) | 76,5% (5) | **46,12% (23)** |
| *Precursors* | *Sulfonamides* | FBSA | 72,8% (4) | 95,9% (1) | 78,5% (4) | 85,8% (1) | 86,5% (1) | **83,9% (8)** |
|  |  | FHxSA | 23,4% (10) | 69,4% (3) | 18,6% (10) | 60,3% (3) | 60,7% (1) | **46,48% (21)** |
|  |  | FOSA | 23,8% (0) | 15% (16) | 8,5% (0) | 52,5% (16) | 25,1% (7) | **24,98% (15)** |
|  | *sulfonamide acetic acids* | L-MeFOSAA | 30,7% (6) | 28,2% (7) | 11,4% (6) | 44,4% (7) | 21,2% (14) | **27,18% (11)** |
|  |  | Br-MeFOSAA | 64,6% (1) | 62,7% (4) | 44,4% (1) | 57,8% (4) | 67,7% (4) | **59,44% (8)** |
|  |  | L-EtFOSAA | 38,6% (5) | 27,3% (4) | 20% (5) | 53,6% (4) | 49,3% (15) | **37,76% (13)** |
|  |  | Br-EtFOSAA | 30,2% (1) | 53,4% (4) | 51,1% (1) | 57,6% (4) | 55,3% (5) | **49,52% (10)** |
|  | *per-/poly-fluoroalkyl ether acids (PFEA)* | ADONA | 1,2% (5) | 16,2% (3) | 4,6% (5) | 18,2% (3) | 18,4% (10) | **11,72% (7)** |
|  |  | 9Cl-PF3ONS | 7,7% (2) | 6% (5) | 6% (2) | 23,6% (5) | 33% (14) | **15,26% (11)** |
|  |  | 11Cl-PF3OUDS | 2,3% (20) | 2,5% (1) | 2,1% (20) | 42,6% (1) | 2,2% (27) | **10,34% (16)** |
|  |  | PF4OPeA | 55,9% (0) | 43,7% (29) | 65,7% (0) | 28,4% (29) | 68,6% (1) | **52,46% (15)** |
|  |  | PF5OHxA | 58,1% (1) | 52,7% (4) | 43,9% (1) | 49,9% (4) | 57,3% (3) | **52,38% (5)** |
|  |  | 3-6-OPFHpA | 69,1% (1) | 64,5% (1) | 65,4% (1) | 78,6% (1) | 74,6% (2) | **70,44% (5)** |
|  |  | PFEESA | 24,8% (5) | 8,8% (5) | 10,4% (5) | 16,3% (5) | 27,4% (4) | **17,54% (7)** |

Reference:

[1] M. Sadia *et al.*, “Occurrence, Fate, and Related Health Risks of PFAS in Raw and Produced Drinking Water,” *Environ. Sci. Technol.*, Feb. 2023, doi: 10.1021/acs.est.2c06015.

[2] H. Sontheimer, J. C. Crittenden, and R. S. Summers, *Activated carbon for water treatment*. 1988.
